# Inferring the physical connectivity of complex networks from their functional dynamics

**DOI:** 10.1186/1752-0509-4-70

**Published:** 2010-05-26

**Authors:** Hung Xuan Ta, Chang No Yoon, Liisa Holm, Seung Kee Han

**Affiliations:** 1 Institute of Biotechnology, PO Box 56, 00014 University of Helsinki, Finland; 2 Department of Physics & BK21 Physics Project, Chungbuk National University, Cheongju, Chungbuk 361-763, Republic of Korea

## Abstract

**Background:**

Biological networks, such as protein-protein interactions, metabolic, signalling, transcription-regulatory networks and neural synapses, are representations of large-scale dynamic systems. The relationship between the network structure and functions remains one of the central problems in current multidisciplinary research. Significant progress has been made toward understanding the implication of topological features for the network dynamics and functions, especially in biological networks. Given observations of a network system's behaviours or measurements of its functional dynamics, what can we conclude of the details of physical connectivity of the underlying structure?

**Results:**

We modelled the network system by employing a scale-free network of coupled phase oscillators. Pairwise phase coherence (PPC) was calculated for all the pairs of oscillators to present functional dynamics induced by the system. At the regime of global incoherence, we observed a Significant pairwise synchronization only between two nodes that are physically connected. Right after the onset of global synchronization, disconnected nodes begin to oscillate in a correlated fashion and the PPC of two nodes, either connected or disconnected, depends on their degrees.

Based on the observation of PPCs, we built a weighted network of synchronization (WNS), an all-to-all functionally connected network where each link is weighted by the PPC of two oscillators at the ends of the link. In the regime of strong coupling, we observed a Significant similarity in the organization of WNSs induced by systems sharing the same substrate network but different configurations of initial phases and intrinsic frequencies of oscillators.

We reconstruct physical network from the WNS by choosing the links whose weights are higher than a given threshold. We observed an optimal reconstruction just before the onset of global synchronization.

Finally, we correlated the topology of the background network to the observed change of the functional activities in the system.

**Conclusions:**

The results presented in this study indicate a strong relationship between the structure and dynamics of complex network systems. As coupling strength increases, synchronization emerges among hub nodes and recruits small-degree nodes. The results show that the onset of global synchronization in the system hinders the reconstruction of an underlying complex structure. Our analysis helps to clarify how the synchronization is achieved in systems of different network topologies.

## Background

Biological networks, such as protein-protein interactions, metabolic, signalling, transcription-regulatory networks and neural synapses, are representations of large-scale dynamic systems. Network maps are graphical representations of dynamic systems in life. A network with a static connectivity is dynamic in the sense that the nodes implement so-called functional activities that evolve in time. In biological context, these activities may represent the concentration of a molecule, phosphorylation state of enzyme, expression level of a gene, depolarization of a neuron or circadian rhythm.

Despite the remarkable diversity of networks in nature, their architecture is governed by a few simple principles that are common to most networks of major scientific and technological interest [1,2]. For decades network systems have been modeled either as chains, grids, lattices and fully-connected graphs which are completely regular or as random Erdős-Rényi network whose node degrees follow a Poisson distribution [3]. However, a number of recent findings indicate that real networks including large communication systems [4-6], biological systems [7-9], and a variety of social interaction structures [10] are characterized by a power-law degree distribution, *P*(*k*) ∝ *k*^-*γ*^, where degree *k *is the number of neighbours of a given node. In these so-called scale-free (SF) networks, most of the nodes have only a few links, whereas a few nodes have a very large number of links, which are often called hubs [11].

Synchronization is a common nonlinear phenomenon in a broad class of systems ranging from physics and chemistry to biology and social sciences [12-14]. This is a dynamical process by which the activities of two or more individuals change coherently, almost in unison. For example, thousands of cells synchronize their activities to make our heart beat rhythmically. An excellent example of a realistic complex network system is the brain, where thousands of neurons fire synchronously to respond to external stimuli. A sudden and unexpected synchronization among a large population of neuronal units may cause some diseases [15], such as epileptic seizures. Therefore, understanding the path to synchronization can be of aid to diagnosing human disease.

Investigating how dynamical activities arise from complex network topology is of fundamental importance to understanding the functions of real-world systems [1,16,17]. Given a model of dynamical elements and the wiring diagram among them, what can we conclude of the dynamics such as synchronization arising from the model? Conversely, how can we infer the presence or absence of individual connections of a network system from observations of its behaviours or measurements of its functional dynamics? For example, how does an electroencephalography (EEG) pattern (which is the recording of electrical activities in different positions within the brain) reflect the details of the axons among cortical neurons?

Most previous studies on structure-dynamics relations analyzed the ability to obtain idealized complete synchronization or collective coherent oscillations, using regular networks with local coupling or in networks with global coupling [13]. It has been shown that the response dynamics of a regular network system when applying external driving signals depends on both the driving and the network connectivity, yielding the ability to reconstruct the network connectivity [18]. Recently, many works have focused on synchronization induced by more realistic complex network systems with the small-world property, the scale-free degree distribution, and the modular hierarchical structure [11,19,20]. The impact of these topological features of a network on the dynamical processes has been intensively studied. For example, it has been shown that the global synchronization is enhanced by the small-world property [21,22] but hindered by the modularity of the network [23-25]. The evolution of global synchronization inside a network system depends much on the heterogeneity of the underlying network [26,27].

In this paper, our aim is to elucidate how the network structure drives the functional dynamics, and consequently, analyze the ability to reconstruct the physical connectivity of an underlying network. We adopt a network of Kuramoto phase oscillators, a useful model to display a large variety of synchronization patterns while being sufficiently exible to be adapted to many realistic systems [28]. The system was investigated comprehensively at both weak and strong regimes of coupling with the background network interpolating between regular and scale-free topologies. The collective behaviour of oscillators in this model is conventionally represented via the global order parameter which is the phase coherence of the population of oscillators [16] (see Methods). Evolution of the global order parameter as a function of coupling strength reflects the path from an incoherent to coherent state of the system. The global order parameter, however, fails to describe where the synchronization emerges and how it propagates inside the system. We calculated the pairwise phase correlation (PPC) for all pairs of oscillators and built a weighted network of synchronization (WNS), an all-to-all functionally connected network where each link is weighted by the PPC of two oscillators at the ends of the link (see Methods). PPC of two phase oscillators shows how dependent their motions are (see Methods). Therefore, the plot of PPC versus the coupling strength *K *and the product of node degrees helps to find which pairs of nodes synchronize first. The changes in the organization of WNS, as the coupling strength increases, help to explore how synchronization is achieved. To be able to observe if the dynamical process is purely driven by the background structure, we compare the WNSs obtained by the systems sharing a common substrate network but different initial configurations.

In spite of dealing with the reconstruction of physical connectivity as the work in [18] does, our work focuses on the reconstruction of physical connectivity from functional dynamics induced by a complex network system without applying any external inputs to the system. Namely, the links are predicted from an averaged WNS based on their weights (see Methods). Whereas the reconstruction of physical connectivity in [18] is implemented only in the stationary state of dynamics with regular background networks, our reconstruction is tested at both regimes of global incoherence and coherence with a family of networks having the same number of nodes and links but different topologies ranging from regular to scale-free. Interestingly, we observed a bimodal distribution of the links weights right before the onset of the global synchronization, irrespective of topologies of the background network. Moreover, the value by which two modes of the distribution are well separated can distinguish very successfully physical links from non-physical links, yielding an optimal reconstruction at this regime of weak coupling. Our findings are useful for practical applications because the weak-coupling regime is biologically more realistic.

## Results and Discussion

### Evolution and Robustness of Synchronization

**Figure 1:**
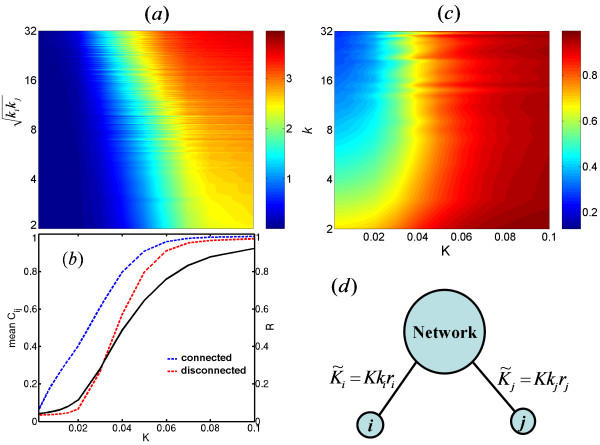
**Evolution of Synchronization inside the system**. (a) Plot of log_10 _(1/(1 - *C*_*ij*_)) versus the degree product *k*_*i*_*k*_*j *_(the data is averaged over all links with the same degree product) and the coupling strength *K*. (b) Evolution of the global order parameter (the black solid curve with the right-hand y-axis) and mean *C*_*ij *_for pairs of connected nodes (the blue dashed curve with the left-hand y-axis) and pairs of disconnected nodes (the red dashed curve with the left-hand y-axis) as a function of *K*. (c) The local order parameter *r *versus the coupling strength *K *and the node degree *k *(the data is averaged over all nodes with the same degree). We used 5 networks and 10 initial configurations for each network. (d) A model of interactions of node *i *and *j*. Node *i *is considered to interact to the network via an effective coupling strength  = *Kk*_*i*_*r*_*i*_.

The critical coupling strength, where the onset of the global synchronization occurs, can be determined by a FSS analysis [29] (see Methods). For the system used in this work, *K*_*c *_≈ 0.02. Figure 1a shows that there is no dependence of PPC between two nodes on their degrees when *K *<*K*_*c*_. In this regime of global incoherence, while a significant pairwise coherence is observed between two nodes that are physically connected (mean physical *C*_*ij *_is approximately 0.3 at *K *= 0.01 and 0.4 at *K *= 0.02), PPC between two disconnected nodes remains close to zero (Figure 1b). Right after the onset of global synchronization, *K *= *K*_*c*_, PPC between two disconnected nodes deviates from zero. PPC of each two nodes begins to depend on their degrees and the PPC of pairs of nodes with the highest  increases first (Figure 1a). PPC for all pairs converges to unity for strong coupling strength, but the monotonic dependence on the product of degrees is still preserved.

**Figure 2:**
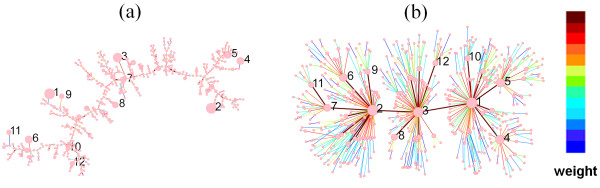
**Visualization of WNSs**. The skeletons of WNS at *K *= 0.002 (a) and *K *= 0.14 (b), respectively. A node is denoted by a circle with its size proportional to the node degree, and the link weight is in the colour scale presented in this figure. The 12 hub nodes with the highest node degrees are labelled.

The organization of synchronization can be visualized by a skeleton network, which depicts the spanning tree (ST) of WNS. Here all nodes are connected as a tree network maximizing the sum of PPC [30]. Figures 2a and 2b are STs of WNSs from the same initial configurations and one background network for coupling strength *K *= 0.002 and *K *= 0.14, respectively. As *K *increases, the structure of the skeleton changes from random to a more defined organization. At the strong coupling, the highest-degree nodes form the backbone of the skeleton. From the core to the periphery of the skeleton graph, the color of links changes from brown to blue, indicating that the synchronization is always the strongest at the core of the skeleton networks but weakens towards the peripheral parts.

The PPC between two nodes depends on the product of their degrees. As the coupling strength increases, the development of the structure of the skeleton of the WNS indicates that synchronization emerges among hub nodes and propagates into peripheral nodes in the system. This phenomenon can be explained as follows. Node *i *is considered to interact with the network via its effective coupling strength,  (Figure 1d). When *K *is strong, *r*_*i *_approaches one (Figure 1c),  is mostly proportional to the degree *k*_*i *_and, thus, hubs have stronger effective coupling. Therefore the hubs are synchronized more easily.

**Figure 3:**
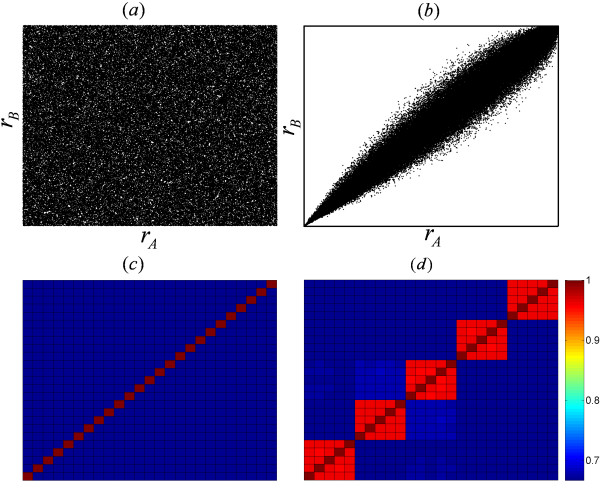
**Robustness of WNSs over Initial Configurations**. Compare the cross link rank in the two WNSs obtained from different initial configurations but the same substrate network at *K *= 0.002 (a) and *K *= 0.140 (b). Plot of the cross link rank preservation *P*_*AB *_for 25 networks (5 different networks and 5 different initial conditions for each network) at *K *= 0.002 (c) and *K *= 0.140 (d).

The robustness of synchronization over initial configurations can be evaluated via the comparison of WNSs obtained with different initial configurations but the same background network. Figures 3a and 3b show the rank of every link in two WNSs, *A *and *B*, at *K *= 0.002 and *K *= 0.140 respectively. It is clear that the link rank is totally random at the weak coupling but highly preserved at the strong coupling. We compute the total cross link rank preservation (CLRP) for 25 WNSs obtained from 5 different substrate networks and 5 different configurations of initial phases and intrinsic frequencies for each substrate network. Unlike Figure 3c for *K *= 0.002, Figure 3d for *K *= 0.140 shows a modular structure; the block-diagonal which represents the intra-group CLPR sharing the same substrate network converges to unity. The off block-diagonal terms which represent the inter-group CLRP with different substrate networks display the random background level of CLRP. The WNSs obtained from the same background network but different initial configuration share similar organization in the synchronized regime of the system. We conclude that the organization of synchronization is independent of the initial configuration and purely driven by the underlying network structure.

### Reconstructing physical connectivity from functional activities

**Figure 4:**
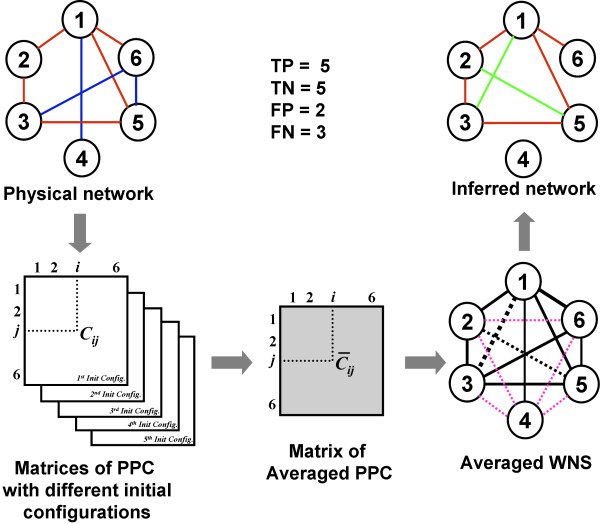
**Schema of the reconstruction of physical connectivity from functional dynamics**. PPC is calculated for all the pairs of nodes with a common underlying physical network but different configurations of initial phases and intrinsic frequencies of oscillators. PPC for all the pairs of nodes is averaged over different initial configurations. Average WNS is built as an all-to-all connected network where each link is weighted by the average PPC between two oscillators at the ends of the links. The solid (dashed) lines denote the physical (non-physical) links which are between nodes connected (disconnected) in the physical network. The inferred network is composed of the links of the WNS whose weights are higher than a given threshold. TP denotes true predicted links (red links in both the physical and inferred networks). FP denotes non-physical links that are predicted as physical links by our method (green links in the inferred network). TN denotes true predicted non-physical links (pink dashed links in the averaged WNS). FN denotes the physical links that are predicted as non-physical links by our method (blue links in the physical network). In this example, TP = 5, TN = 5, FP = 2 and FN = 3.

Predicting the underlying structure of a system from its observed behaviour is an important task in many fields. Here, we focus on finding which regime of coupling facilitates the reconstruction of physical connectivity from functional activities. The procedure of reconstruction is depicted in Figure 4.

**Figure 5:**
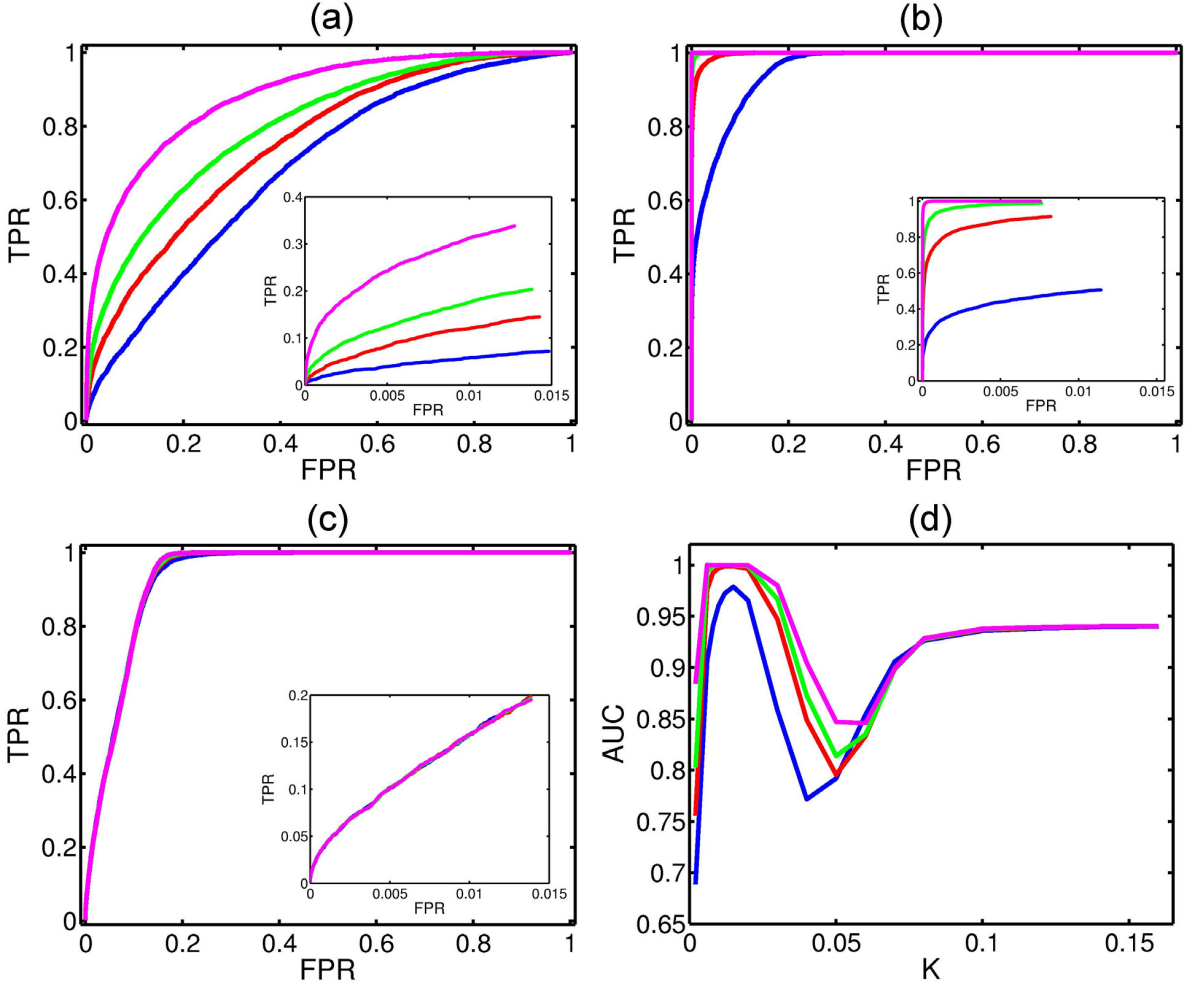
**Reconstruction of Underlying Struture from WNS**. ROC curves to depict the reconstruction of physical connectivity with one (blue), three (red), five (green) and ten (magenta) initial configuration(s) as the coupling strength *K *= 0.002 (a), *K *= 0.010 (b) and *K *= 0.100 (c). The insets in (a-c) are the corresponding ROC curves up to the first 2000 predicted links. Plotting AUC versus *K *helps to find the optimal regime of coupling strength for reconstruction of physical connectivity (d).

The reconstruction of physical connectivity is implemented at several values of coupling strength. Figure 5 shows the performance of the reconstruction of physical network with one, three, five and ten initial configurations at *K *= 0.002 (Figure 5a), *K *= 0.010 (Figure 5b) and *K *= 0.100 (Figure 5c). The corresponding insets present ROC curves up to the first 2000 predicted links. The results indicate that averaging over many initial configurations enhances the reconstruction of physical connectivity. This is due to the fact that averaging the functional measurement of a system over many trials helps to reduce the noise existing inside the system and, thus, helps to represent the dynamical process in the system more precisely [31].

**Figure 6:**
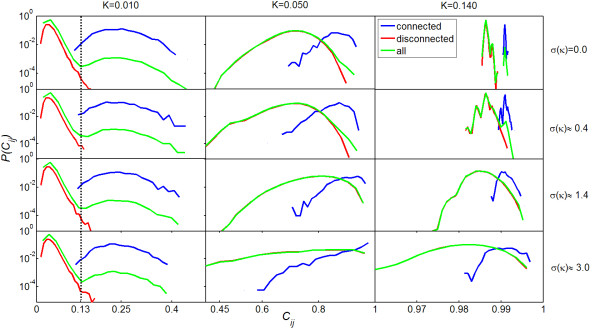
**Distribution of PPCs**. Distribution of PPC for physically connected nodes (blue), disconnected nodes (red) and all pairs of nodes (green) at different coupling strength and for different network topologies. The data is averaged over five different initial configurations. At *K *= 0.010 a bimodal distribution of PPCs for all pairs of nodes is observed in all the network topologies. The threshold used to reconstruct physical network can be around 0.130 by which two modes of the distribution are well separated.

The optimal reconstruction is observed from *K *= 0.01 to 0.02 (Figure 5d), before the initiation of synchronization, irrespective of how many initial configurations are used. This suggests that the functional dynamics right before the onset of synchronization have a close relationship with the underlying network topology. In this regime of coupling strength, we observed a bimodal distribution of all links in terms of their weights (see Figure 6 for *K *= 0.010 and *σ*(*κ*) ≈ 3.0). We suggest setting the threshold approximately equal to 0.13 by which two different modes are well separated. Around this regime of global incoherence, *K *= 0.01, there occurs a significant pairwise correlation among pairs of nodes that are directly (physically) connected [26,27]. Moreover, due to the fact that the propagation of signals inside the system remains limited at a weak coupling, there is no significant local correlation among disconnected nodes. Therefore, physical links dominate over non-physical links in terms of PPC. In the regime of global coherence, the signal can propagate through the whole network; this corresponds to pathological situations such as epileptic seizures [32]. In this regime, the oscillators at hub nodes start to oscillate in a correlated fashion even though these nodes are not physically connected. Therefore, both physical and non-physical links can exist in the top weighted links of WNS. While the synchronization emerges and grows in the system, the reconstruction of the background network becomes weaker. We conclude that the onset of synchronization hinders the inference of physical connectivity of complex networks from the functional dynamics.

### Impact of heterogeneity of substrate network on synchronization

**Figure 7:**
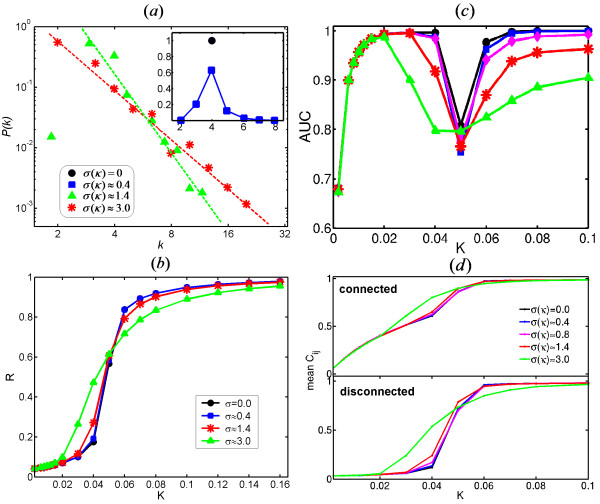
**Impact of Underlying Network Topology on Synchronization**. (a) Degree distribution of networks. (b) Evolution of global order parameter as a function of coupling strength for different network topologies corresponding to *σ*(*κ*) = 0 (regular), 0.4, 1.4 and 3.0 (SF). (c) Plot of AUC, used to quantify the performance of reconstruction, versus *K *with *σ*(*κ*) = 0 (black), 0.4 (blue), 0.8 (magenta), 1.4 (red) and 3.0 (green). (d) Evolution of the mean PPC for physically connected nodes (upper panel) and disconnected nodes (below panel) as a function of *K*.

To understand the influence of the heterogeneity of network structures on the synchronization, we studied a family of static random networks satisfying the degree distribution *P*(*k*) ∝ *k*^-*γ *^*e*^-*k*/*κ *^with a controllable exponential cut-off scale *κ *[33]. Many real-world graphs show this exponential cut-off in the degree distribution [10,34]. In these networks, the variance in the degree distribution, *σ*(*κ*), varies as a function of *κ *while the mean degree remains constant. *σ*(*κ*) measures the degree of heterogeneity of the networks in that *σ*(*κ*) = 0 corresponds to regular network, *σ*(*κ*) ≈ 0.4 to homogeneous network and *σ*(*κ*) ≈ 3.0 to heterogeneous SF network (Figure 7a). In [27], the authors also used a family of networks interpolating between homogeneous and heterogeneous topologies to study the impact of topology on the synchronization transition.

Figure 7b shows the evolution of the global order parameter as a function of coupling strength *K *for several network topologies ranging from regular to scale-free. The onset of global synchronization first occurs for the SF network. The more heterogeneous the network is, the smaller value of *K *is needed for the onset of the global synchronization. Conversely, the path to the complete synchronization is faster for networks with a homogeneous degree distribution. Whereas the nodes in homogenous network system suddenly become globally synchronized as the coupling strength increases, the path to synchronization of heterogeneous network system is nontrivial: synchronization starts among hubs and recruits low-degree nodes.

Figure 7d shows the behaviour of PPC for physically connected nodes (upper panel) and disconnected nodes (lower panel) in different types of networks. In the regime of global incoherence (*K *<*K*_*c*_), for all network topologies, the mean PPC of physically connected nodes is Significant whereas that of two disconnected nodes is approximately zero, indicating that the dynamical process in the systems at a weak coupling is driven by physical connectivity. To clarify this point, we show the distribution of all links in terms of their weights in Figure 6. In this regime of weak coupling, we observed a bimodal distribution where the right-side (left-side) mode is well-consistent with the distribution of physical (non-physical) links, irrespective of network topologies. Namely, the value that separates two modes of the distribution can distinguish very successfully physical links from non-physical links. Therefore, reconstruction of an underlying network from the functional activities is highly possible for all network topologies, at this weak coupling regime (Figure 7c).

Right at the onset of global synchronization (*K *≈ 0.02 for *σ*(*κ*) ≈ 3.0, 0.03 for *σ*(*κ*) ≈ 1.4 and 0.04 for *σ*(*κ*) = 0.0), mean PPC for disconnected nodes deviates from zero for all network topologies (lower panel, Figure 7d), indicating that pairwise correlation between disconnected nodes starts at the emergence of global synchronization. Moreover, this happens earlier in more heterogeneous networks. In Figure 6, as *σ*(*κ*) increases, the overlap between two PPC distributions for connected and disconnected nodes at *K *= 0.05 becomes more and more significant. The distribution of all links shows a unimodal distribution, causing a difficulty to determine the threshold while implementing the reconstruction of physical connectivity. This is due to the fact that in heterogeneous networks, such as SF network, disconnected hub nodes are strongly pairwise synchronized whereas some connected small-degree nodes remain weakly correlated. This dependence of the PPC of two nodes on their degree is shown in Figure 1a. The existence of pairwise synchronization between the nodes that are not physically connected hinders the inference of a background network from the functional activities around *K *= 0.05 for all network topologies (Figure 7c).

At the strong coupling, *K *= 0.140, the behaviour of PPC changes when the network topology varies between homogeneous and heterogeneous (Figure 6). In homogeneous networks (*σ*(*κ*) = 0.0 and *σ*(*κ*) ≈ 0.4), both of the distributions of PPC for connected and disconnected nodes are very sharp compared to those in heterogeneous networks. This indicates that, at a strong coupling strength, PPC of two nodes in homogeneous networks purely depends on whether they are physically connected or not. Only for homogeneous networks does the distribution of all links show a bimodal distribution, facilitating the inference of the underlying network for homogeneous topology at the strong coupling (Figure 7c). In heterogeneous networks, the more neighbours a node has, the higher effective coupling strength via which it interacts with the network. This results in the dependence of the PPC of two nodes on their degrees. At a strong coupling strength, hub nodes are strongly pairwise synchronized whether they are connected or disconnected. In heterogeneous networks, the distributions of PPC both for connected and disconnected nodes are broader, and there is a Significant overlap between the two populations. This results in a lower capacity for reconstructing the background network for heterogeneous networks in the regime of strong coupling.

## Conclusions

There have been many works aiming to discover the relationship between the dynamical process in the system and the underlying physical connectivity [21-27]. Many studies performed so far have investigated the impact of background network topologies on the collective behaviour of the system as represented by the global order parameter [23-27,35]. In this paper, we have comprehensively investigated a dynamical network system at both regimes of weak and strong coupling with a family background networks interpolating between regular and scale-free topologies.

Our study on the collective behaviour of the system proves that heterogeneous network systems are easier to be synchronized whereas homogeneous network systems go to the complete synchronization faster. By calculating pairwise phase correlation for every pair of either connected or disconnected nodes, we showed how the synchronization emerges and propagates inside the system. The homogeneous distribution of node degree results in the fact that the pairwise correlation of two nodes in a homogeneous network is purely dependent on whether they are connected or not. In heterogeneous network systems, the path to synchronization of heterogeneous network system is nontrivial. Synchronization starts among hubs and propagates toward low-degree nodes.

The strong relationship between the physical connectivity and the functional dynamics suggests an ability to solve one of inverse problems: reconstruct physical connectivity from observed functional dynamics. In spite of dealing a similar reconstruction of physical network, we use the synchronization pattern induced by a complex network system without external inputs whereas Timme M. in [18] use the method of analyzing the response dynamics of the network system upon perturbation. More comprehensively, our reconstruction of physical connectivity was tested for the systems with network topologies ranging from regular to complex at both weak and strong regime of coupling. We pointed out that the regimes of weak coupling, right before the onset of global synchronization, facilitate a successful reconstruction of physical connectivity, irrespective of network topologies. Clearly, our method to reconstruct physical connectivity is applicable to a wide range of networks from regular to scale-free topologies.

Our study of functional dynamics in complex networks has important implications for medicine. The sudden emergence of synchronization among the elements of a system might cause serious damages. For example, excessive synchronization of neuronal activity in basal ganglia cortical loops is the hallmark of activity in Parkinson's disease [36]. An epileptic seizure is assumed to be associated with abnormal excessive or synchronous neuronal activity in the brain [37]. If we know, which locations inside the system become synchronized first, we can anticipate the systems' damages prior to their occurrence.

## Methods

### System Modelling

We start with a Barabási and Albert (BA) network of 512 nodes grown by using the preferential attachment rule [19], which has a scale-free degree distribution *P*(*k*) ∝ *k*^-*γ *^with the scaling exponent *γ *≈ 3 and the mean degree  = 4. We adopt a Kuramoto model of non-identical phase oscillators [13] in the network system. Therefore, the differential equations modelling the system are(1)

where *i *= 1, ..., *N*, *θ*_*i *_and *ω*_*i *_are the phase initiated randomly within [-*π*, *π*] and the intrinsic frequency distributed uniformly within [-0.1, 0.1] of node *i*, *K *is the coupling strength. To make the model more realistic, we add Gaussian white noise, *ξ*_*i*_(*t*) with intensity *D *satisfying ⟨*ξ*_*i*_(*t*)⟩ = 0 and ⟨*ξ*_*i*_(*t*)*ξ*_*j*_(*t'*)⟩ = 2*Dδ*_*ij *_*δ*(*t - t'*). In this study, we use *D *= 0.01. *A*_*ij *_is an element of the adjacency matrix, which is 1 if nodes *i *and *j *are connected and 0 otherwise. The coupling term, therefore, is summed over the neighbours of node *i*. We solved the eqs. (1) by using the improved Euler method with a step size of 0.01.

### Global Order Parameter

The collective behavior of the oscillators system is conventionally represented by the global order parameter defined as(2)

where the brackets ⟨...⟩_*t *_signify the time averaging. The global order parameter measures the extent of global synchronization of the population of *N *oscillators.

We calculate the global order parameter at different regimes of coupling strength to study how the collective behaviour of all the oscillators changes from full-desynchronized and full-synchronized states.

### Finite Size Scaling Analysis

Finite Size Scaling (FSS) analysis is used to determine the critical coupling strength separating desynchronized and synchronized states. FSS analysis used in this work follows the procedure presented in [29]. The work in [38] uses the same technique for a finite-size scaling analysis. The global order parameter is described in terms of a scaling function as(3)

where *F *is the scaling function, *N *is the size of network, *β *and *ν *are the critical exponents. The function *F *has a unique value, which is independent of *N*, at *K *= *K*_*c*_. The derivative of *R *at *K*_*c *_satisfies the following constraint(4)

Plotting *RN*^*β*/*ν *^versus *K *for various network size, one can find the value of *β*/*ν *that gives a well-defined crossing point at *K*_*c*_. In this work, *N *= 128, 256, 512 and 1024. After obtaining *K*_*c *_and *β*/*ν*, one can use eq. (4) to determine the value of (1- *β*)/*ν*, which combined with the known value of *β*/*ν*, yields the values of *β *and *ν*.

### Pairwise Phase Coherence

The local synchronization between two phase oscillator *i *and *j*, either connected (physical connection) or disconnected (non-physical connection), is quantified by pairwise phase coherence which is defined as follow.(5)

*C*_*ij*_, which shows how dependent the motions of two oscillators at nodes *i *and *j *are, is equal to 0 or 1 corresponding the full incoherence or coherence between nodes *i *and *j*, respectively. This measure of phase coherence was used in [27].

We monitored the changes in PPC of all pairs of nodes as *K *increases to find which pairs of nodes synchronized first. In Figure 1a, we plot log (1/(1 - *C*_*ij*_)) versus the coupling strength *K *and the product of *k*_*i *_and *k*_*j *_to see how the PPC depends on the neighborhood connectivity. The range of color from blue to brown corresponds to the value of *C*_*ij *_changing from 0 to nearly 1.

### Weighted Network of Synchronization

We define a weighted network of synchronization as an all-to-all functionally connected network where each link is weighted by PPC of two oscillators at the ends of the link. In a WNS, physical and non-physical links are between the nodes that are connected and disconnected in the background network, respectively. WNS, therefore, keeps the information of the organization of synchronization of the system.

### Local Order Parameter and Effective Coupling Strength

The governing eqs. (1) can be rewritten as(6)

where(7)

In (7), *r*_*i *_is the local order parameter which measures the coherence of *k*_*i *_neighbors of node *i*. The oscillator at node *i *interacts with the network via an effective coupling strength  = *Kk*_*i*_*r*_*i *_(Figure 1d).

### Cross Link Rank Preservation

CLRP is used to compare two weighted networks. All links of each network are ranked according to their weights. Let  be the rank of link *α *in the network *A*(*B*). CLRP of *α *over network *A *and *B *is quantified by , where *L *is the total number of links in a WNS. The total CLRP between two WNSs *A *and *B *is averaged over all links in the WNSs.(8)

### Reconstruction of Physical Connectivity

The reconstruction of physical connectivity from functional dynamics is depicted in Figure 4. The eqs. (1) are solved with different configurations of initial phases and intrinsic frequencies of oscillators. A so-called averaged WNS is built where each link is weighted by the PPC averaged over several initial configurations. In the averaged WNS, links whose weights are higher than a given threshold are predicted as physical links. On the contrary, predicted non-physical links have weights that are lower than the threshold.

The threshold can be determined by presenting the distribution of all links in terms of their weights. At the regimes of weak coupling where the reconstruction performs the best, the weight distribution of all links shows a bimodal distribution. The value by which two modes of the distribution are well separated can be used as the threshold. For example, at *K *= 0.010, a bimodal distribution of all links is observed for all the network topologies and the threshold can be around 0.130 (Figure 6).

The performance of reconstruction is analyzed by a Receiver Operating Characteristic (ROC) curve, which depicts relative trade-offs between true positive (benefits) and false positive (costs). We compute true positives (TP), false positives (FP), true negatives (TN) and false negatives (FN). TP denotes true predicted links. FP denotes non-physical links that are predicted as physical links by our method. TN denotes true predicted non-physical links. FN denotes the physical links that are predicted as non-physical links by our method. In the plot of the ROC curve, the *x*-axis represents false positive rate (FPR), that is FP/(TN+FP), and the *y*-axis represents true positive rate (TPR), TP/(TP+FN) as the threshold gradually varies.

In this study, the best reconstruction would yield a point in the upper left corner or coordinate (0,1) of the ROC space, representing 100% true predicted physical links without false positives. The (0,1) point corresponds to an optimal reconstruction. It the physical network was reconstructed by randomly choosing links from the WNS, this would give a point along a diagonal line from the left bottom to the top right corners.

The reconstruction performance is quantified by the area under the ROC curve (AUC), which is usually used for model comparison in machine learning [39]. This measure can be interpreted as the probability that when we randomly pick one positive and one negative example, the classifier will assign a higher score to the positive example than to the negative. An AUC of 0.5 reflects a reconstruction of physical network by randomly choosing links from the WNS, while AUC = 1 implies a perfect reconstruction.

## Authors' contributions

HXT and SKH initiated the work. HXT carried out the mathematical research and implemented the numerical simulations with guidance from SKH and support of CNY for programming. The manuscript was written by HXT and revised by LH for important intellectual content. All co-authors have approved the final version.
